# miR‐24 and its target gene Prdx6 regulate viability and senescence of myogenic progenitors during aging

**DOI:** 10.1111/acel.13475

**Published:** 2021-09-24

**Authors:** Ana Soriano‐Arroquia, John Gostage, Qin Xia, David Bardell, Rachel McCormick, Eugene McCloskey, Ilaria Bellantuono, Peter Clegg, Brian McDonagh, Katarzyna Goljanek‐Whysall

**Affiliations:** ^1^ Institute of Life Course and Medical Sciences University of Liverpool Liverpool UK; ^2^ The Medical Research Council/Versus Arthritis Centre for Integrated Research into Musculoskeletal Aging CIMA University of Liverpool Liverpool UK; ^3^ Discipline of Physiology School of Medicine National University of Ireland Galway Ireland; ^4^ Department of Oncology and Metabolism Healthy Lifespan Institute and the Centre for Integrated Research in Musculoskeletal Aging University of Sheffield Sheffield UK

**Keywords:** aging, miR‐24, muscle regeneration, oxidative stress, Prdx6, satellite cells, senescence

## Abstract

Satellite cell‐dependent skeletal muscle regeneration declines during aging. Disruptions within the satellite cells and their niche, together with alterations in the myofibrillar environment, contribute to age‐related dysfunction and defective muscle regeneration. In this study, we demonstrated an age‐related decline in satellite cell viability and myogenic potential and an increase in ROS and cellular senescence. We detected a transient upregulation of miR‐24 in regenerating muscle from adult mice and downregulation of miR‐24 during muscle regeneration in old mice. FACS‐sorted satellite cells were characterized by decreased levels of miR‐24 and a concomitant increase in expression of its target: Prdx6. Using GFP reporter constructs, we demonstrated that miR‐24 directly binds to its predicted site within Prdx6 mRNA. Subtle changes in Prdx6 levels following changes in miR‐24 expression indicate miR‐24 plays a role in fine‐tuning Prdx6 expression. Changes in miR‐24 and Prdx6 levels were associated with altered mitochondrial ROS generation, increase in the DNA damage marker: phosphorylated‐H2Ax and changes in viability, senescence, and myogenic potential of myogenic progenitors from mice and humans. The effects of miR‐24 were more pronounced in myogenic progenitors from old mice, suggesting a context‐dependent role of miR‐24 in these cells, with miR‐24 downregulation likely a part of a compensatory response to declining satellite cell function during aging. We propose that downregulation of miR‐24 and subsequent upregulation of Prdx6 in muscle of old mice following injury are an adaptive response to aging, to maintain satellite cell viability and myogenic potential through regulation of mitochondrial ROS and DNA damage pathways.

## Graphical abstract

Age‐related changes in miR‐24 and its target gene Prdx6 contribute to defective function of myogenic progenitors and muscle regeneration during aging. miR‐24 upregulation and downregulation of its target Prdx6 is associated with an increase in mitochondrial ROS, increase in pH2Ax, decreased cell viability, myogenic potential and increased senescence. During aging, downregulation of miR‐24 in satellite cells and after muscle injury may represent a compensatory mechanism acting to preserve cell viability and myogenic potential

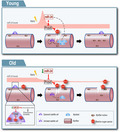

**Abbreviations**
BaCl_2_
: barium chloride
CM‐H2‐DCFDA: chloromethyl derivative of 2',7'‐dichlorodihydrofluorescein diacetate
FACS: Fluorescence‐activated cell sorting
GAS: gastrocnemius
H2Ax: histone H2AX
miR: microRNA
miRNA: microRNA
p16: cyclin‐dependent kinase inhibitor 2A
p21: Cyclin Dependent Kinase Inhibitor 1A
Prdx6: Peroxiredoxin 6
ROS: reactive oxygen species
SA‐βgal: senescence‐associated beta galactosidase
TA: tibialis anterior
WGA: wheat germ agglutinin

## 1 INTRODUCTION

The regenerative capacity of skeletal muscle facilitates a high plasticity for adaptation to diverse metabolic conditions and energetic demands. Skeletal muscle regeneration after injury and loading stressors relies on satellite cells, the adult muscle stem cells able to regenerate muscle fibers in vivo. A balance between satellite cells self‐renewal and myogenic differentiation is essential for successful muscle regeneration after injury (Sambasivan & Tajbakhsh, 2015). However, the effectiveness of muscle regeneration throughout lifespan not only relies on the functionality of satellite cells (Lepper et al., 2011); disrupted intracellular signaling and an altered muscle fiber microenvironment are known to play a key role in muscle wasting during disuse, aging, and chronic diseases (Fry et al., 2015; Le Moal et al., 2017). In particular, oxidative stress has been demonstrated to alter the cellular microenvironment, resulting in disrupted cellular signaling and potentially oxidative modifications of muscle contractile proteins (Goljanek‐Whysall et al., 2016; Sakellariou et al., 2017). Some of the important muscle antioxidant proteins that directly affect intracellular ROS concentrations are members of the peroxiredoxin family: (PRDX1‐PRDX6). Peroxiredoxins have the capacity to regulate redox homeostasis and signaling pathways involved in processes such as apoptosis and cell survival or in response to injury. Particularly, peroxiredoxin 6 (Prdx6) has been demonstrated to regulate both myogenesis and adipogenesis via the control of glucose uptake (Pacifici et al., 2014; Wu et al., 2015), and *Prdx6* ^−/−^ mice display increased levels of markers of senescence, metabolic sarcopenia, and loss of muscle strength (Pacifici et al., 2020).

microRNAs (miRNAs, miRs) are short non‐coding RNAs approximately 20–22 nucleotides in length. miRs show partial complementarity to their target mRNA(s) and regulate gene expression at the post‐transcriptional level (Lee et al., 1993). miRs are known to regulate a myriad of biological processes, including muscle homeostasis and aging through processes such as ROS generation and scavenging (Goljanek‐Whysall et al., 2020). miR‐24 is highly expressed in skeletal muscle (Wada et al., 2011) and has been proposed to regulate myogenesis in vitro and to inhibit muscle fibrosis in vivo (Sun et al., 2008, 2018). Yet, the functional role of miR‐24 in human and mouse primary myogenic stem/progenitor cells, including oxidative stress, and in skeletal muscle aging remains elusive.

In this study, we identified changes in miR‐24:Prdx6 interactions in satellite cells during aging. Our data confirm a decline in satellite cell fraction, viability, and myogenic potential in muscle from old mice. miR‐24 expression was downregulated in FACS‐sorted satellite cells during aging, with the concomitant upregulation of its target Prdx6. Our results demonstrate a transient upregulation of miR‐24 in regenerating muscle from adult mice after acute injury, whereas in old mice, we detected downregulation of miR‐24 expression during muscle regeneration. Using GFP reporter constructs, we demonstrated the binding of miR‐24 to its target site within Prdx6 mRNA. Changes in miR‐24:Prdx6 interactions were associated with altered mitochondrial ROS generation and levels of phosphorylated‐H2Ax in myogenic progenitors and affected their viability, myogenic potential, and senescence. The effects of miR‐24 up‐ and downregulation were more pronounced in myogenic progenitors from old mice, suggesting a context‐dependent role of miR‐24 in these cells. We propose that changes in miR‐24:Prdx6 interactions during aging are aimed at preserving satellite cells viability and function. We hypothesize that age‐related downregulation of miR‐24 and subsequent increased Prdx6 expression in satellite cells represents an adaptive mechanism aimed to improve the regenerative capacity of skeletal muscle through preserving satellite cell viability and function by regulating ROS‐associated pathways.

## 2 RESULTS

### 2.1 miR‐24 is downregulated during skeletal muscle regeneration and aging

**FIGURE 1:**
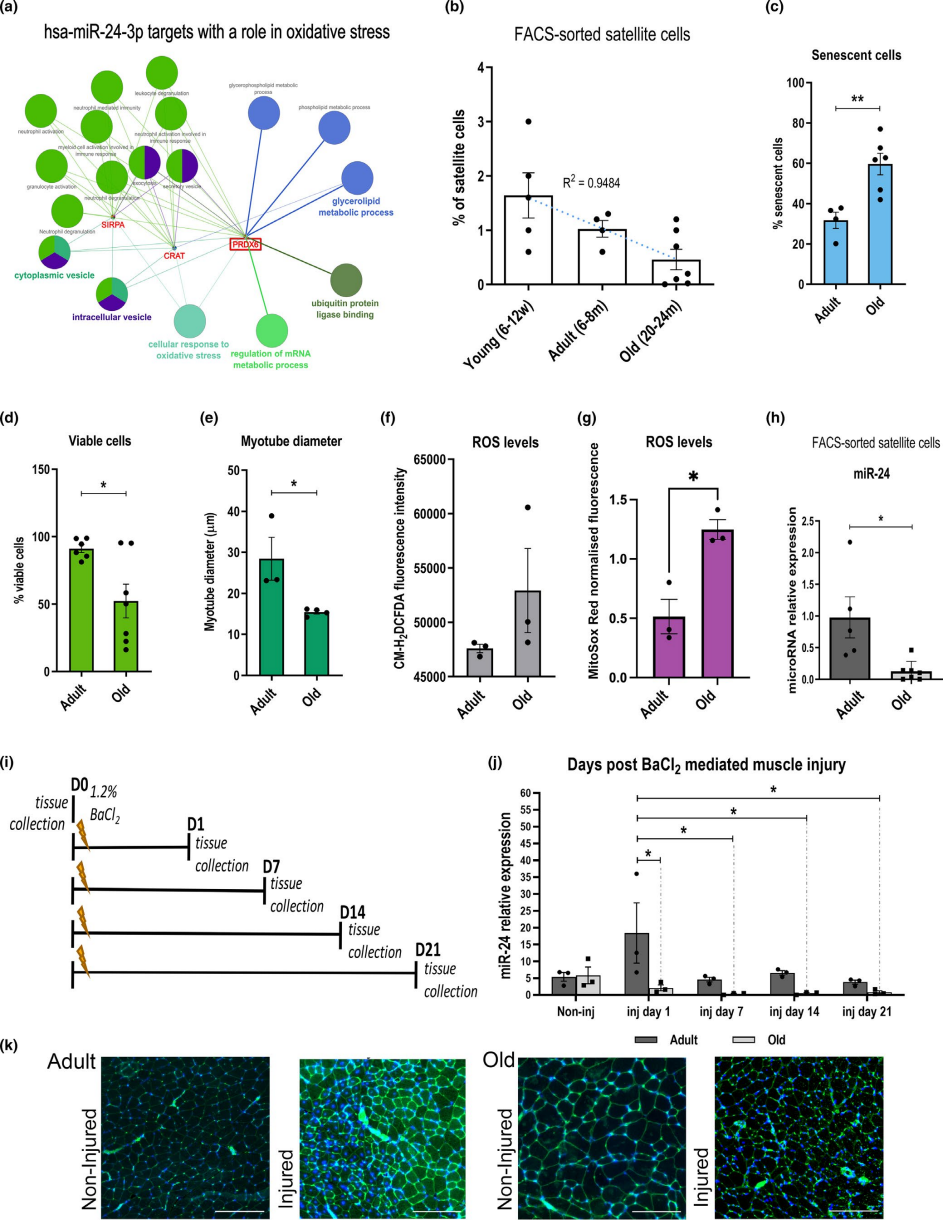
miR‐24 expression is affected by muscle injury and aging. (a) miR‐24 is predicted to target genes and processes associated with redox balance in humans. Gene ontology (GO) analysis was performed by ClueGO plugin for Cytoscape (v.2.5.6). Statistical test used for ClueGO: enrichment/depletion (two‐sided hypergeometric test). *p*‐value cutoff =1.0E‐4. Correction method =Bonferroni step down. Min GO level =5; max GO level =8; number of genes =16; min percentage =4.0; Kappa score threshold =0.4. Only targets involved in “cellular response to oxidative stress” are shown. (b) The percentage of mouse satellite cells decreases during aging (*n* = 4–7, *R* ^2^ = 0.9484). (c–e) Myogenic progenitors from old mice are less viable, have decreased myogenic potential, and display increased senescence (*n* = 3–6, two‐tailed unpaired Student's *t*‐test). (f) The accumulation of ROS assessed using the CM‐H_2_DCFDA assay; mean fluorescence intensity shown. (g) Increased production of mitochondrial ROS detected by MitoSox Red in mouse myogenic progenitors during aging (*n* = 3, unpaired *t* test). (h) qPCR showing decreased expression of miR‐24 in mouse satellite cells during aging. Expression relative to Snord61 is shown (*n* = 5–7, Mann–Whitney test). (i) Diagram representing tissue collection points following TA injury using BaCl_2_. (j) qPCR of miR‐24 in the TA after injury. Expression relative to Snord61 is shown (*n* = 3, two‐way ANOVA followed by Tukey's multiple comparison test with 95% confidence interval). Young: 6–12 weeks old; adult: 6–8 months old; old: 20–24 months old. (k) Representative images of WGA and DAPI staining indicating the extent of muscle damage following BaCl_2_ injury of the gastrocnemius muscle from adult and old muscle. Scale bars: 500 µm. For miR‐24 qPCR in satellite cells: adult: 1–8 months old; old: 20–24 months old. *p*‐value < 0.05 was considered as statistically significant (*). Error bars show S.E.M

**FIGURE 2:**
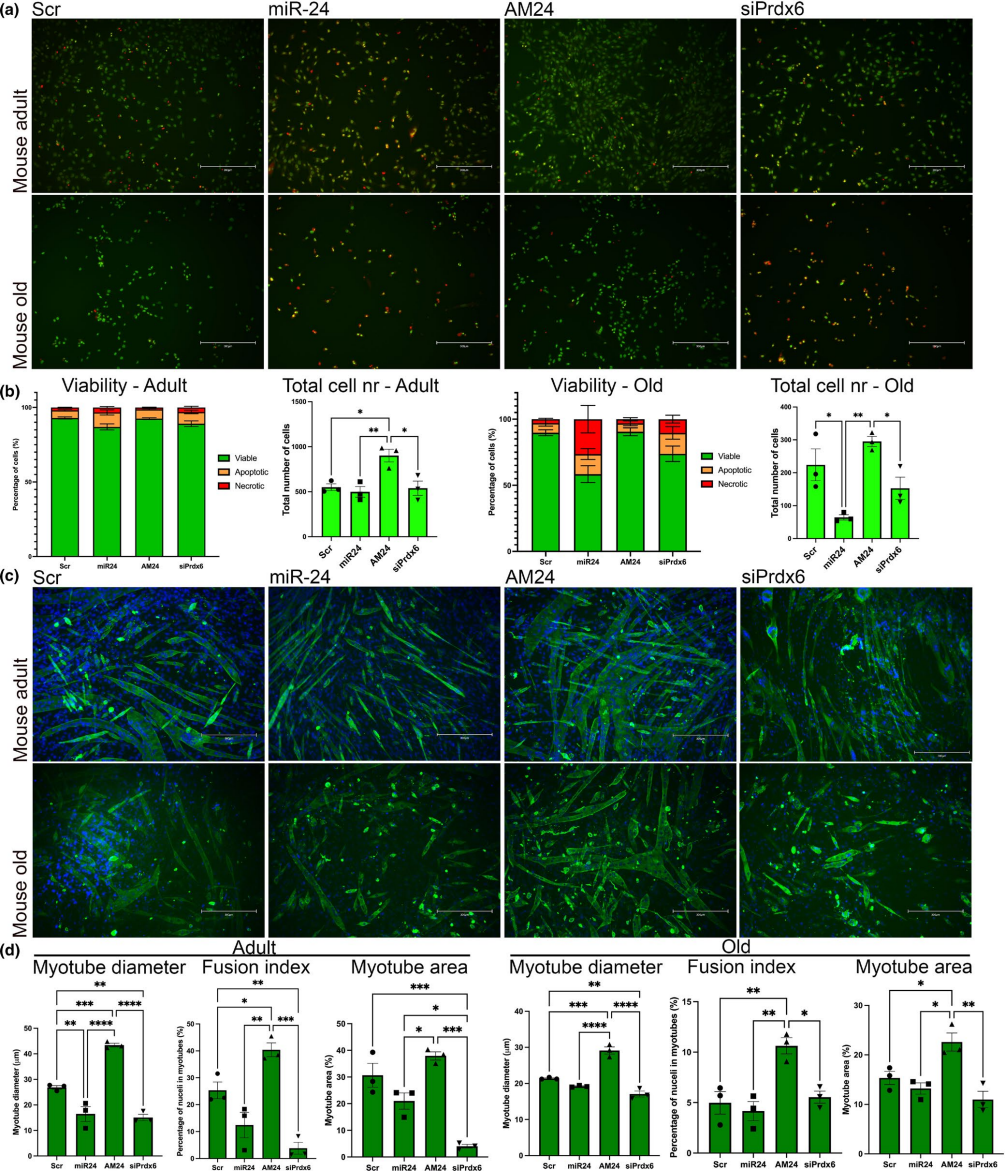
miR‐24 regulates viability and differentiation of myogenic progenitors during aging. (a and d) Myogenic progenitors isolated from adult and old mice were transfected with miR‐24 or AM24. Cells transfected with scrambled control were used as control. Scale bars: 300 µm. (a) Viability assay shows viable (green), apoptotic (yellow), and necrotic (red) cells. (b) miR‐24 overexpression and downregulation of its target Prdx6 resulted in a significant decrease in % viable cells from old mice. (c) MF 20 (antimyosin heavy chain; green) and DAPI (blue) immunostaining were performed for myogenic differentiation and nuclei identification, respectively. Scale bars: 300 µm. (d) Overexpression of miR‐24 significantly affected the differentiation of myogenic progenitors from adult mice, inhibition of miR‐24 target Prdx6 inhibited myogenic differentiation of muscle progenitors from muscle of adult and old mice, whereas miR‐24 inhibition led to improved myogenesis of muscle progenitors from muscle of adult and old mice. All panels: *n* = 3–4, one‐way ANOVA followed by Tukey's multiple comparison test. Adult: 6 months old; old: 24 months old. *p*‐value < 0.05 was considered as statistically significant (*p < 0.05). Error bars show S.E.M

**FIGURE 3:**
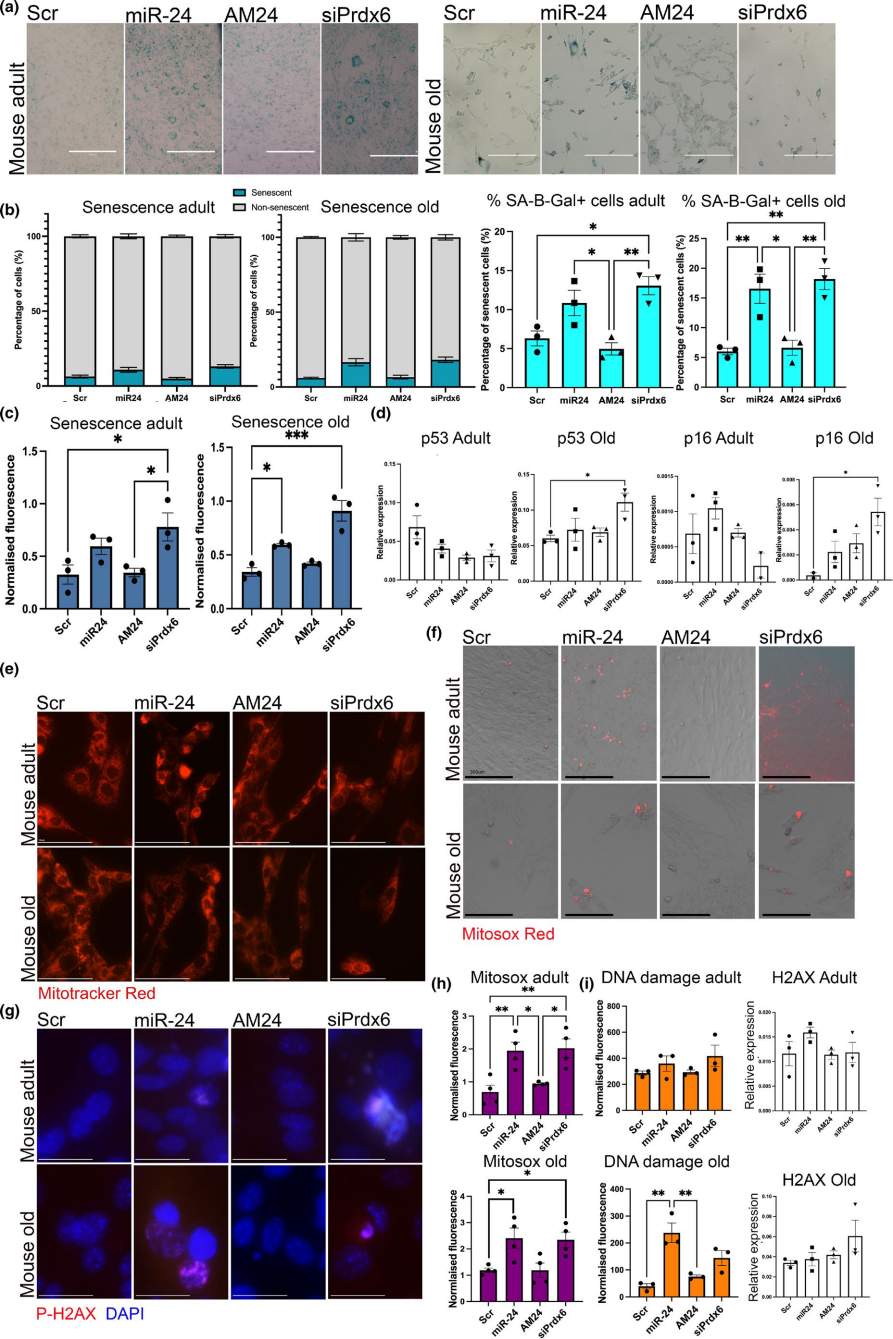
miR‐24 overexpression and downregulation of its target gene Prdx6 lead to increased number of senescent cells through increased mitochondrial ROS generation and DNA damage. (a) Myogenic progenitors isolated were transfected with miR‐24 mimic (miR‐24) or anti‐miR (AM24) and transfected at P7, early senescence stage. Cells transfected with scrambled control were used as control. SA‐βgal staining (b) or fluorescent SA‐βgal staining (c) was performed for the assessment of senescent cells (blue). Scale bars: 300 µm (b and c) miR‐24 overexpression and inhibition of Prdx3 resulted in a higher % of senescent cells in the adult or both mice, respectively; *n* = 3, one‐way ANOVA followed by Tukey's). (d) miR‐24 and Prdx6 overexpression/inhibition was associated with increased p53 and p16 expression in myogenic progenitors from old mice; expression relative to 29S is shown; one‐way ANOVA followed by Tukey's. (e) MitoTracker staining of myogenic progenitors from adult and old mice indicates dysregulation of mitochondrial networks following miR‐24 overexpression and Prdx6 downregulation. Scale bars: 75 µm. (f) MitoSox Red staining indicates increase in mitochondrial ROS production following miR‐24 overexpression and Prdx6 downregulation. Scale bars: 300 µm. (g) Overexpression of miR‐24 and inhibition of Prdx6 led to an increase in the presence of nuclei stained for phosphorylated H2Ax, a marker of DNA damage. Scale bars: 30 µm (h) Quantification of MitoSox Red staining and phosphor‐H2Ax staining indicate increase in ROS generation following miR‐24 overexpression and Prdx6 downregulation in myogenic progenitors from adult and old mice and increase in DNA damage marker in muscle of old mice following miR‐24 overexpression. miR‐24 and siPrdx6 expression manipulation did not result in changes in H2ax expression (qPCR). Adult: 6 months old; old: 24 months old. *p*‐value < 0.05 was considered as statistically significant (*p < 0.05;). Error bars show S.E.M

**FIGURE 4:**
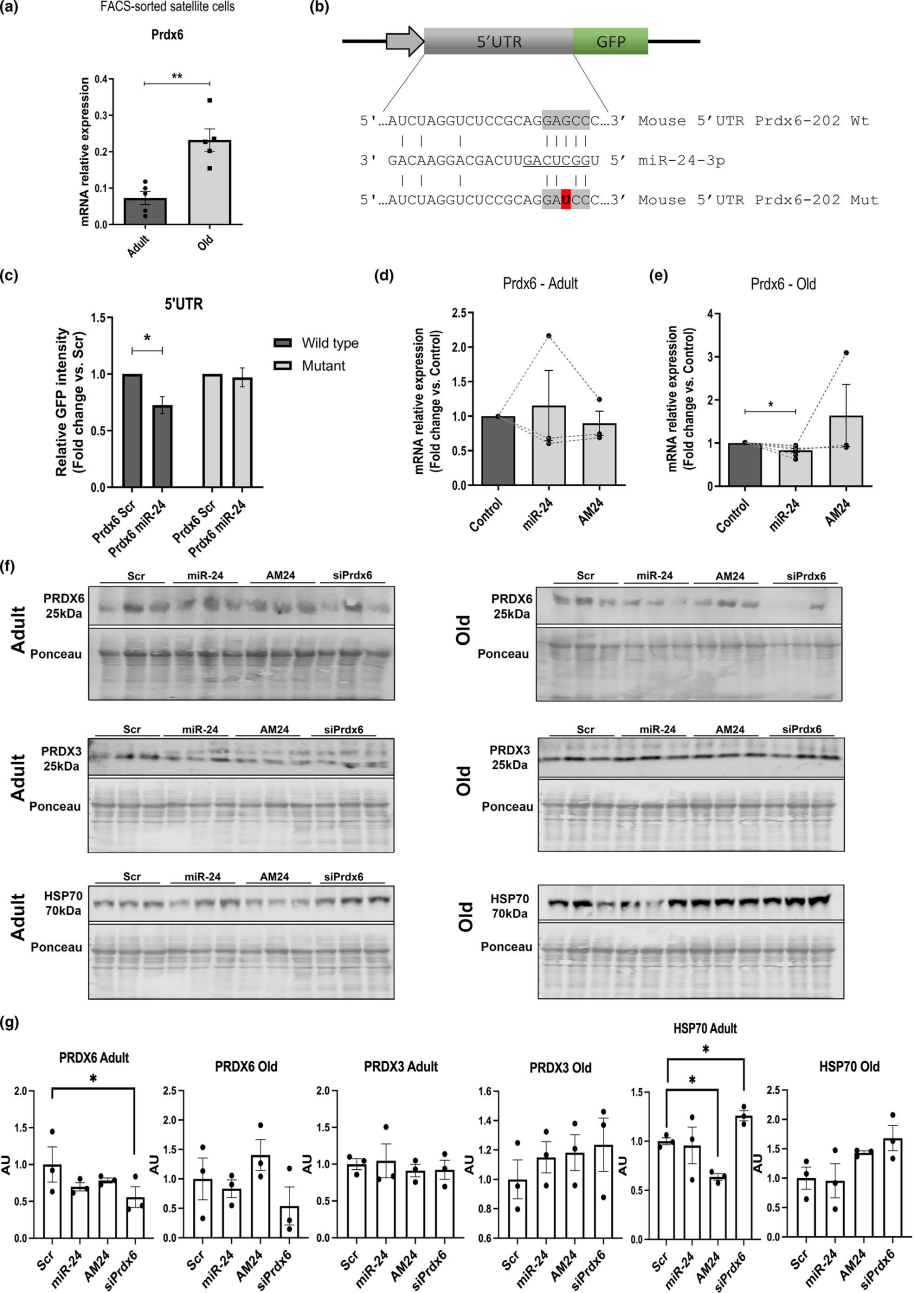
miR‐24 fine‐tunes the levels of Prdx6 levels through its target site. (a) qPCR showing Prdx6 expression in mouse satellite cells during aging. Expression relative to beta‐actin is shown. Adult: 1–8 months old. Old: 20–24 months old (*n* = 5, Mann–Whitney test). (b) Putative miR‐24‐3p seed sequence in the 5’ UTR of Prdx6 gene (highlighted in gray). Mutated seed sequence used for 5’UTR microRNA:mRNA target interaction is shown. Mutation is shown in red. (c) miR‐24 directly regulates the expression of Prdx6. GFP‐Prdx6 5’UTR sensor construct containing the wild‐type or mutated seed sequence was transfected into C2C12 myoblast cell line and co‐transfected with miR‐24 or scrambled control (Scr). The wild‐type construct transfected with miR‐24 mimic shows less GFP fluorescence intensity compared to the scrambled control, but not in the mutated construct (representative data shown; *n* = 3, two‐tailed unpaired Student's *t* test). (d and e) qPCR showing the expression of Prdx6 after microRNA mimic or antagomiR (AM24) transfection in primary myogenic progenitors isolated from adult (d) and old mice (e). Expression relative to 18S is shown. Adult: 6–8 months old; old: 20–24 months old (*n* = 3–7, Kruskal–Wallis test followed by Dunn's multiple comparisons test with 95% confidence interval). (f and g) Western blotting indicating changes in PRDX6 levels following miR‐24 overexpression or inhibition suggesting miR‐24 fine‐tuning the levels of PRDX6 rather than being a master regulator of PRDX6 levels. miR‐24 overexpression and inhibition or downregulation of PRDX6 had no effect on the levels of antioxidant protein PRDX3, *n* = 3. One‐way ANOVA followed by Tukey's multiple comparison. For all the figures unless stated otherwise: adult: 6 months old; old: 24 months old. *p*‐value < 0.05 was considered as statistically significant (*p < 0.05). Error bars show S.E.M

miR‐24 has been previously shown to be regulated during satellite cell activation (Cheung et al., 2012; Redshaw et al., 2014). Some of the miR‐24 putative targets in humans, analyzed using TargetScan and ClueGO plugin for Cytoscape, were genes associated with the cellular response to oxidative stress, including Prdx6 (Figure 1a). We focused on miR‐24 target genes associated regulation of viability, differentiation, and senescence through regulation of redox balance, as redox homeostasis has been shown to regulate all these processes during aging (Le Moal et al., 2017). Prdx6 has been shown to regulate skeletal muscle adaptation under increased oxidative stress (Da Silva‐Azevedo et al., 2009). Prdx6 was upregulated in FACS‐sorted satellite cells isolated from old mice compared to adult mice (Figure 4a), but not in the tibialis anterior muscle from old mice (Figure S2). This is consistent with downregulation of miR‐24 expression in satellite cells but not muscle during aging (Figure 1h, j). We therefore investigated age‐related changes in miR‐24:Prdx6 interactions in satellite cells. We observed a decrease in the total number of FACS‐sorted satellite cells during aging (Figure 1b, Figure S1), consistent with previously published data (Gibson & Schultz, 1983). Myogenic progenitors from old mice displayed increased senescence (Figures 1c, 3a), reduced viability (Figures 1d, 2a), reduced myogenic potential (Figures 1e, 2d), and increased ROS (Figure 1f, g). miR‐24 expression was downregulated in satellite cells from old mice (Figure 1h). The expression of miR‐24 was also analyzed by RT‐qPCR in an in vivo model of skeletal muscle regeneration following barium chloride (BaCl_2_) injection (Figure 1i–k). Local muscle injury with BaCl_2_ has been shown to induce depolarization of the sarcolemma, membrane rupture, proteolysis, and motor denervation in the skeletal muscle fibers (Morton et al., 2019). However, BaCl2‐induced muscle damage preserves satellite cells allowing for detailed study of their role in muscle regeneration (Morton et al., 2019). Most of the damage resolved by 21 days in adult but not old mice, in which central nuclei remain after 21 days (Figure 1k). miR‐24 basal levels were not altered in TA muscle during aging, as opposed to its downregulation in satellite cells (Figure 1h, j), but its expression was increased one day after muscle injury and returned to basal levels after seven days in the injured muscle of adult mice, suggesting a potential role of miR‐24 in the early stages of muscle regeneration after acute injury. However, the expression of miR‐24 did not increase in old mice following injury: miR‐24 expression was significantly lower at 1–21 days after injury compared to the adult mice (Figure 1j). These data suggest that the downregulation of miR‐24 in satellite cells (Figure 1h) may be related to the age‐related decline in satellite function and consequently muscle regeneration following acute injury.

### 2.2 miR‐24 regulates viability and myogenic potential of myogenic progenitors during aging

The function of satellite cells in muscle regeneration depends on their viability and myogenic potential, and both are affected by aging (Figure 1d, e). To determine the physiological consequences of miR‐24 and its target Prdx6 dysregulation in satellite cells during aging and regeneration, myogenic progenitors isolated from adult and old mice were transfected with miR‐24 mimic, AM24, siRNA for Prdx6 or scrambled RNA (control) and stained to evaluate differentiation (MF 20), proliferation (Ki67), and viability (Figure 2 and Figure S4). miR‐24 had no significant effect on myogenic progenitor proliferation (Figure S3). However, overexpression of miR‐24 and downregulation of Prdx6 expression resulted in the increased proportion of necrotic and apoptotic myogenic progenitors from adult and old mice and inhibition of miR‐24 resulted in an increase in the total number and number of viable myogenic progenitors (Figure 2a, b). Moreover, overexpression of miR‐24 and downregulation of its target: Prdx6, in myogenic progenitors from adult and old mice, resulted in inhibition of myogenesis (Figure 2c, d). These data were consistent with previous, independently performed analyses of miR‐24 role in myogenic progenitors (Figure S3).

### 2.3 miR‐24 and its target Prdx6 regulate senescence of myogenic progenitors

Satellite cells have been previously shown to undergo senescence during aging (Blau et al., 2015; Zhu et al., 2019). Senescent cells become present in both culture from adult and old myogenic progenitors at passage 7. Cells were then transfected with miR‐24 mimic or inhibitor (AM24) or Prdx6 siRNA. Overexpression of miR‐24 or Prdx6 downregulation led to a higher proportion of senescent cells in myogenic progenitors from both adult and old mice, as well as higher number of senescent cells as measured by SA‐βgal staining and by measurement of fluorescent SA‐βgal (Figure 3a‐c). These differences were not significant when highly senescent (over 60% cells positive for SA‐βgal) myogenic progenitors from adult and old mice were treated with miR‐24 or AM24; this could indicate that miR‐24 can regulate only the early stages of senescence but also is likely associated with cycle context‐dependent role of miR‐24 (Figure S3C, see also Discussion). The expression of senescence‐associated genes p16 and p53 was not changed in myogenic progenitors from adult mice following miR‐24 overexpression or Prdx6 downregulation (Figure 3d, Figure S3E). In myogenic progenitors from old mice, miR‐24 overexpression and downregulation of Prdx6 led to increased p16 and p53 levels, which were significantly different in cells treated with siRNA for Prdx6 (Figure 3d). Our initial experiments in highly senescent myogenic progenitors from mice suggested downregulation p16, p21, and p53 in myogenic progenitors from old mice, which could be due to miR‐24 directly targeting these genes rather than it regulating senescence at later/irreversible stages of cellular senescence (Figure S3E, see also Discussion). Inhibition of miR‐24 in myogenic progenitors from old mice had no significant effect on number of SA‐βgal positive cells or the overall proportion of senescent cells or expression of senescence‐associated genes (Figure 3a‐d). This could be associated with already low levels of miR‐24 in cells from older mice or altered levels of other target genes of miR‐24, such as p21 in myogenic progenitors from adult and old mice (Lal et al., 2009) (Mishra et al., 2009) (Lu et al., 2018). The latter is supported by our initial assessment of the role of miR‐24 in regulating senescence of myogenic progenitors, where miR‐24 had different effects on changes in senescence‐associated gene expression in highly senescent cells from adult and old mice, despite similar effects on cellular senescence on the phenotypic level (Figure S3C,E).

### 2.4 miR‐24 and its target Prdx6 regulate mitochondrial ROS production and the levels of DNA damage marker

As Prdx6 has been previously shown to regulate mitochondrial dynamics and function, myogenic progenitors cells transfected with miR‐24, AM24, or Prdx6 siRNA and stained with MitoTracker Red to visualize mitochondria (Figure 3e), as well as MitoSox Red and phosphorylated H2Ax, to detect mitochondrial ROS and DNA damage, respectively (Figure 3e‐h). Myogenic progenitors from both adult and old mice showed disrupted mitochondrial morphology, increased mitochondrial ROS production, and increase in DNA damage marker following overexpression of miR‐24 or downregulation of Prdx6 expression (Figure 3e‐h). These effects were more pronounced in myogenic progenitors from old mice, consistent with the differences in regulation of senescence by miR‐24 in myogenic progenitors from adult and old mice, suggesting a context‐dependent function of miR‐24. Together, these data indicate a potential mechanism of regulation of myogenic progenitor senescence and viability by miR‐24 and its target Prdx6, where increased levels of miR‐24 and concomitant downregulation of Prdx6 lead to disruption of mitochondrial morphology, increase in mitochondrial ROS production, increase in DNA damage marker levels, and induction of pro‐apoptotic and/or pro‐senescent pathways, likely through p16 and p53 signaling.

### 2.5 miR‐24 directly regulates the expression of Prdx6 in myogenic progenitors

Prdx6 is a confirmed miR‐24 target in human cells (Li et al., 2016). We next analyzed the sequence of mouse Prdx6 for miR‐24 binding sites. A target site for miR‐24 was found between position 2–6 (5‐mer) of the mature microRNA‐24 and position 41–45 5’UTR of the mouse Prdx6‐202 transcript (Figure 4b). A GFP reporter containing the wild‐type or mutated miR‐24 binding site for miR‐24 was generated (Figure 4c). C2C12 myoblasts were transfected with reporter constructs containing wild‐type or mutated miR‐24 binding site within the Prdx6 5’UTR fragment and co‐transfected with miR‐24 mimic or scrambled sequence (control). GFP levels were decreased in the cells transfected with wild‐type construct co‐transfected with miR‐24 as compared to scrambled treated cells, but not in the cells treated with the mutated construct co‐transfected with miR‐24 or control Scr microRNA. These results confirm that miR‐24 directly binds to Prdx6 mRNA in mouse myoblasts (Figure 4c). We next investigated Prdx6 expression following miR‐24 overexpression or downregulation in myogenic progenitors from adult and old mice. Prdx6 expression was significantly downregulated following miR‐24 overexpression in myogenic progenitors from old but not adult mice (Figure 4e). Similarly, protein levels of PRDX6 were affected by miR‐24 levels in old but not adult mice, although these changes did not reach statistical significance, likely due to relatively small *n* number (*n* = 3). Cell treated with Prdx6 siRNA showed lower levels of PRDX6, although the level of downregulation varied between individual replicates (Figure 3f,g). Interestingly, changes in the levels of miR‐24 or Prdx6 did not affect the levels of a mitochondrial peroxiredoxin PRDX3. However, inhibition of miR‐24 or downregulation of Prdx6 levels resulted in changes in the levels of chaperone protein HSP70 in adult progenitors suggesting a potential mechanism underlying the phenotypic differences between the effects of miR‐24 on myogenic progenitors from adult and old mice (Figure 4f,g).

### 2.6 miR‐24 regulation of myogenic potential and viability by controlling Prdx6 is conserved in human myogenic progenitors

**FIGURE 5:**
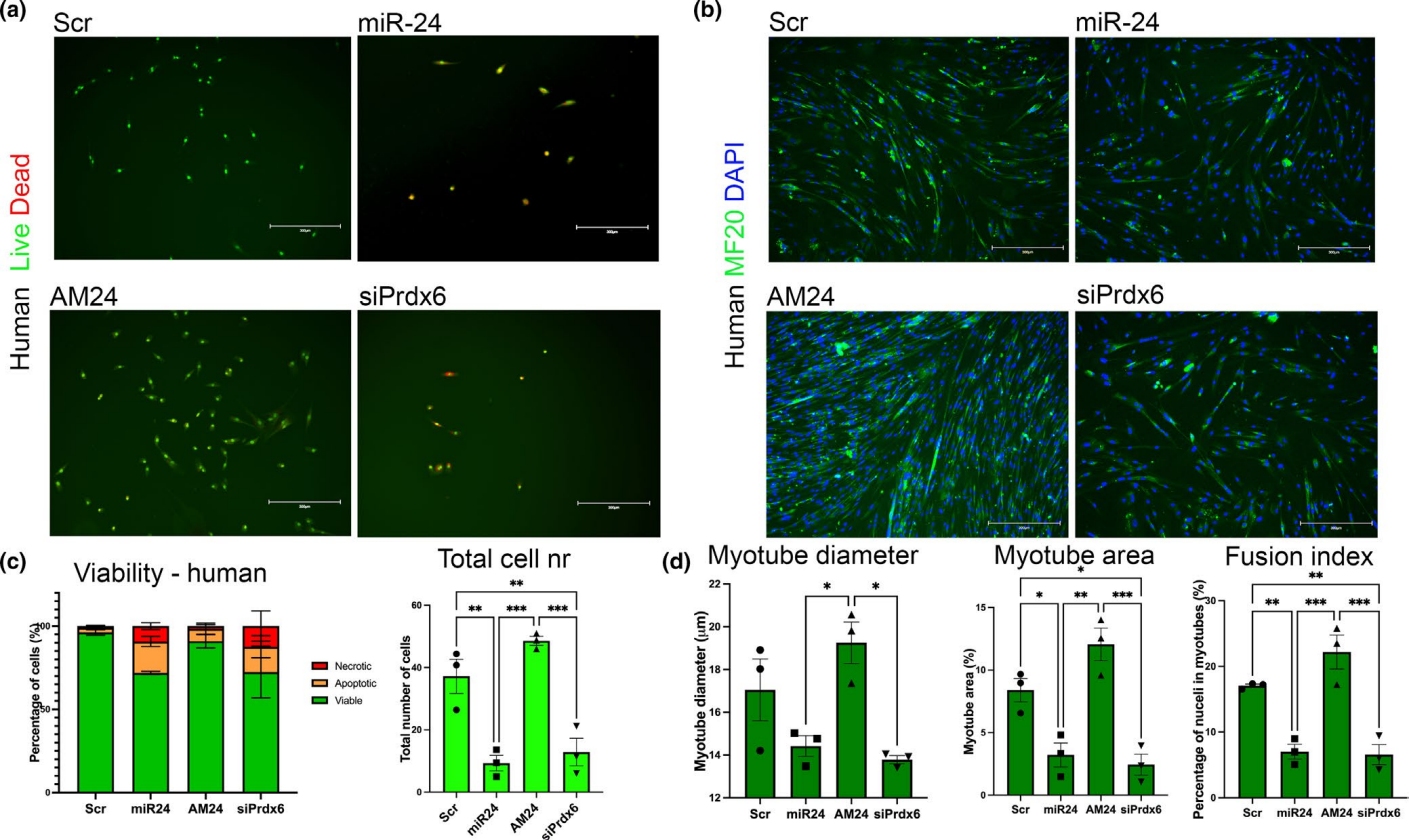
miR‐24 and Prdx6 regulate viability and differentiation of human primary myoblasts. (a and b) Human primary myogenic progenitor cells isolated from adults were transfected with miR‐24 or AM24 or siRNA for Prdx6. Cells transfected with scrambled RNA were used as control (control). MF 20 (antimyosin heavy chain; green) and DAPI (blue) immunostaining were performed for myogenic differentiation and nuclei identification, respectively. Viability assay was performed with ethidium bromide and acridine orange for the assessment of viable (green), apoptotic (yellow), and necrotic (red) cells. SA‐βgal staining was performed for the assessment of senescent cells (blue). Scale bars: 200 µm. (c and d) miR‐24 overexpression and downregulation of Prdx6 resulted in decreased number of viable cells, as well as thinner myotubes containing fewer nuclei (*n* = 3, one‐way ANOVA followed by Tukey's multiple comparison). *p*‐value < 0.05 was considered as statistically significant (*p < 0.05). Error bars show S.E.M. Scale bars: 300 µm

We further explored whether the function of miR‐24 and its target Prdx6 is conserved in human cells. Myogenic progenitors isolated from adults were transfected with miR‐24 mimic, AM24, siRNA for Prdx6, or scrambled control. miR‐24 overexpression and downregulation of Prdx6 expression resulted in decreased cell viability and lower total cell number as well as decreased myogenic potential with miR‐24 overexpression and siPrdx6 downregulation resulting in the presence of smaller myotubes containing fewer nuclei (Figure 5a‐d). miR‐24 had no effect on proliferation of human myogenic progenitors (Figure S4), consistently with the lack of miR‐24 regulation of murine cell proliferation. These data are consistent with murine data and our initial assessment of miR‐24 role in human myogenic progenitors (Figure S4A,B).

### 2.7 miR‐24 and its target Prdx6 regulate mitochondrial ROS production and senescence of human progenitors.

**FIGURE 6:**
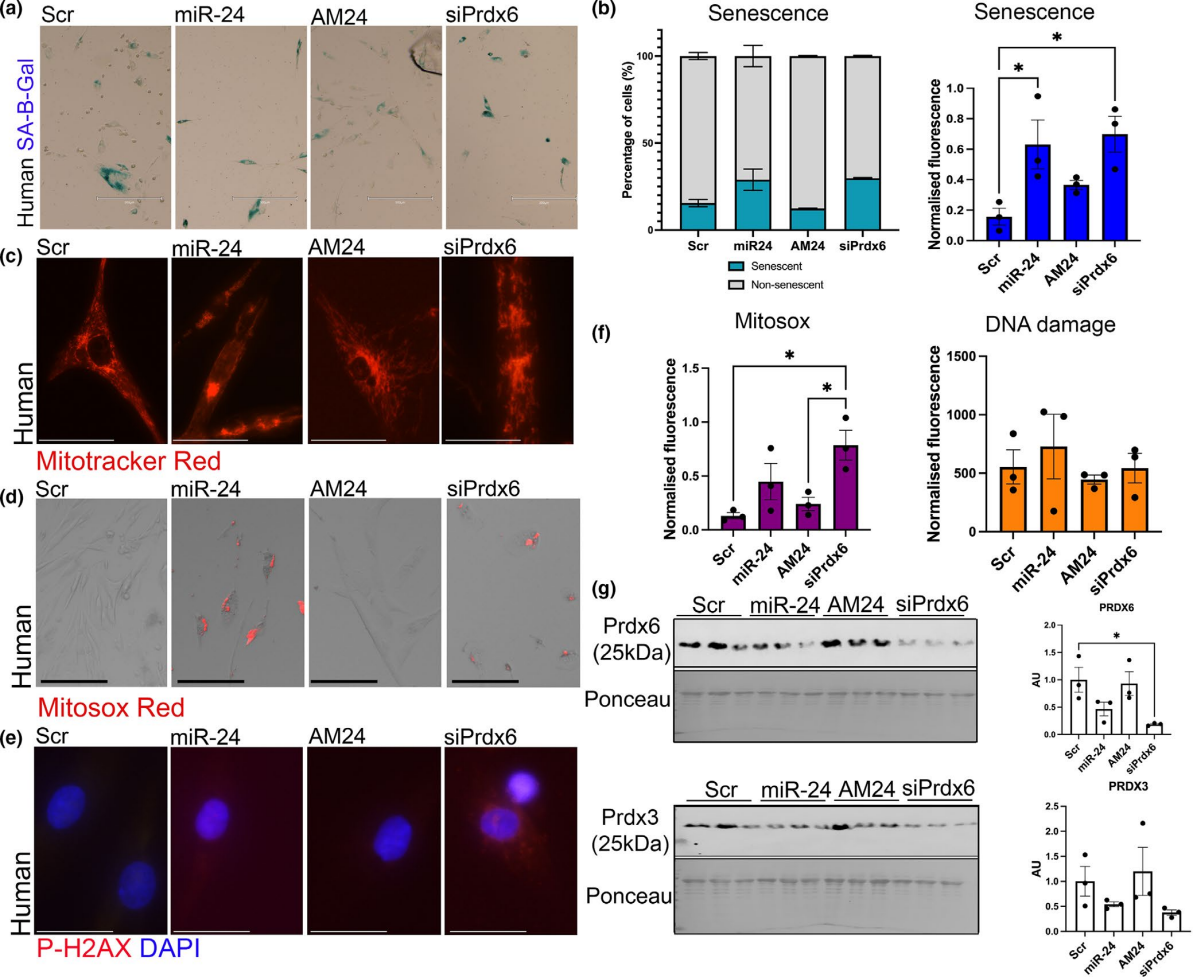
miR‐24 and its target regulate senescence of human myogenic progenitors through mitochondrial ROS production. (a) Human myogenic progenitors isolated were transfected with miR‐24 mimic (miR‐24) or anti‐miR (AM24). Cells transfected with scrambled control were used as control. SA‐βgal staining (b) or fluorescent SA‐βgal staining (c) was performed for the assessment of senescent cells (blue). Scale bars: 300 µm. (b) Quantification of senescent cells indicates increase in the proportion and number of senescent cells following miR‐24 overexpression and downregulation of Prdx6 expression. (c) MitoTracker Red staining indicates changes in mitochondrial morphology following miR‐24 overexpression and downregulation of siPrdx6 levels. (d and f) MitoSox Red staining indicates increased mitochondrial ROS levels following miR‐24 overexpression and downregulation of Prdx6 expression. Scale bars: 75 µm. (e and f) No significant changes were detected in DNA damage marker, phosphorylated H2Ax. Scale bars: 30 µm. (g) Western blot indicating miR‐24 fine‐tuning the expression of Prdx6 rather than being a master regulator of its expression. No changes were detected in the levels of antioxidant protein levels: PRDX3 following changes in miR‐24 or Prdx6 expression. One‐way ANOVA, followed by Tukey's multiple comparison test with 95% confidence interval. *p*‐value < 0.05 was considered as statistically significant (*p < 0.05). Error bars show S.E.M

miR‐24 overexpression and downregulation of Prdx6 expression both led to an increased number of senescent cells (Figure 6a, b) as compared to control group. To determine whether miR‐24 and Prdx6 were involved in regulating ROS levels in human cells, primary myogenic progenitors were transfected with miR‐24 mimic, anti‐miR (AM24), siRNA against Prdx6 (siPrdx6), or scrambled control RNA (Scr) (Figure 6). Similar to the results obtained from myogenic progenitors isolated from mice (Figure 4), disrupted mitochondrial morphology and increased mitochondrial ROS production were detected after miR‐24 overexpression and Prdx6 silencing in comparison with scrambled control group (Figure 6c‐f). In addition, miR‐24 overexpression and Prdx6 silencing led to the presence of nuclei positive for phosphorylated H2Ax, a marker of DNA damage; however, this increase did not reach statistical significance (Figure 6e,f). Western blot analyses revealed downregulation of PRDX6 levels following miR‐24 overexpression (not significant) and Prdx6 siRNA (significant); however, no changes were detected in the levels of antioxidant protein PRDX3 (Figure 6g). Together, these data indicate that miR‐24 and its target Prdx6 regulate the viability, senescence, and myogenic potential through controlling pathways associated with mitochondrial ROS generation, DNA damage, and potentially unfolded protein response (UPR) with the different effects of miR‐24 and Prdx6 on the levels of HSP70 in myogenic progenitors from adult and old mice, indicating a potential mechanism underlying the stronger effects of miR‐24 on cells from old mice.

## 3 DISCUSSION

Muscle aging is associated with the disruption of a wide range of physiological processes affecting the myocyte niche, compromising satellite cell functionality and their regenerative potential in response to injury (Sannicandro et al., 2019).

Following injury, an acute increase in endogenous ROS is required to promote a pro‐inflammatory environment that helps with macrophage recruitment (Horn et al., 2017). ROS levels decrease at later stages of regeneration to allow muscle hypertrophy and remodeling (Laumonier & Menetrey, 2016). However, this process must be tightly regulated, as chronically elevated ROS may induce irreversible protein modifications, aberrant signaling, DNA damage, and mutagenesis (Kidane et al., 2014). When damage persists, cellular stressors can trigger a transient cell cycle arrest via activation of p53/p21 or p16/pRB axes, which can eventually result in the induction of cellular senescence or cell death programs such as apoptosis and autophagy (Vicencio et al., 2008).

This study aimed to investigate the underlying biological mechanisms of the microRNA miR‐24‐3p and its target gene Prdx6 in muscle regeneration during aging. Our results demonstrate a transient increase in miR‐24 expression one day after acute injury in an in vivo model of skeletal muscle regeneration in adult mice. miR‐24 expression returned to baseline levels 7 days after injury, when myoblasts stop proliferating and start differentiating to initiate tissue remodeling in mice (Grounds, 2014). Similar to the results presented here, miR‐24 expression is dynamically changed during gastric metastasis progression (Li et al., 2016). We have also identified an upregulation of the antioxidant Prdx6 in mouse quiescent satellite cells during aging and confirmed Prdx6 as a direct target gene of miR‐24 in mice. However, changes in Prdx6 mRNA and protein levels were modest following miR‐24 overexpression and inhibition (Figure 4), suggesting a fine‐tuning rather than major regulator role of miR‐24 in controlling the levels of PRDX6.

Our results suggest that downregulation of miR‐24 in satellite cells and concomitant upregulation of its target gene Prdx6 are associated with disrupted mitochondrial network morphology, increased ROS generation, an increase in the levels of phosphorylated‐H2AX, a marker of DNA damage, and on a phenotypic level, a decrease in cellular viability and myogenic potential, as well as increase in senescence of surviving cells. Interestingly, miR‐24 has been previously shown to regulate senescence‐associated genes (Lal et al., 2009) (Mishra et al., 2009) (Lu et al., 2018), and our results also showed changes in the expression of p16, p21, and p53 in primary myogenic progenitors after miR‐24 overexpression or Prdx6 downregulation (Figure 3d, Figure S3). While on a phenotypic level, changes in miR‐24 levels consistently regulated cellular senescence, some differences were observed in miR‐24 regulation of senescence‐associate genes, including miR‐24 target genes: p16, p21, between cells which have or not undergone replicative senescence in culture (Figure 3, Figure S3) and cells from adult and old mice (Figure 3, Figure S3). The effects of Prdx6 downregulation on cell senescence were consistent in all cells, suggesting that miR‐24 may regulate cellular senescence through multiple targets in addition to Prdx6. For example, miR‐24 has been shown to increase DNA damage through regulating the levels of H2AX protein or p21 (Lal et al., 2009). Moreover, according to published data, miR‐24 regulates the expression of tumor suppressor/senescence‐associated proteins differently depending on the cell type and metabolic state of the cell: It reduced p16 protein levels in human diploid fibroblasts and cervical carcinoma cells or highly senescent cells (Figure S3), and it inhibited H2Ax in terminally differentiated hematopoietic cells making them vulnerable to DNA damage (Lal et al., 2009). On the other hand, miR‐24 has been shown to increase p53 and p21 protein levels in different cancer cell lines (Mishra et al., 2009) and to induce p53 expression in human epithelial cells during aging and oxidative stress (Lu et al., 2018). Together, these results suggest that miR‐24 exerts either an inhibitory or enhancer function over tumor suppressor/senescence‐associated proteins depending on the cell cycle state, which is consistent with our findings: miR‐24 acted as an enhancer of senescence at early senescent stages (Figure 3) and, however, did not further increase the proportion of senescent cells in population of highly senescent cells (Figure S3). Context‐dependent role of miRs has been previously demonstrated, as well as their dose‐dependent regulation of physiological processes (Vasudevan, 2012), and warrants careful examination of cell properties when investigating microRNAs or other potential senolytics. Moreover, miR‐24 may regulate the expression of senescence‐regulated genes *via* an upstream regulatory factor not yet identified by us. The delicate balance between apoptotic, antiapoptotic, proliferative, and cell cycle arrest signals will ultimately determine whether some cells successfully differentiate/self‐renew or, in contrast, die/become senescent.

Moreover, the diverse activities of Prdx6, including peroxidase, PLA2 phospholipase, and LPCAT activities, mean that it could potentially regulate different metabolic signaling pathways, from cell cycle, membrane repair, and antioxidant response (Arevalo & Vázquez‐Medina, 2018; López Grueso et al., 2019). One limitation of this manuscript is that it did not explore the function of Prdx6 as PLA2 phospholipase in the context of muscle repair and aging.

Another limitation of this study is the variability in the efficiency of the transfection experiments. For the qPCR experiments, only samples where transfection efficiency was validated, either by an increased or inhibited expression of miR‐24, were taken into consideration for the quantification of the target gene expression. In particular, the inhibition of miR‐24 was challenging to achieve despite using two different miR‐24 inhibitors, which resulted in a reduced number of the independent replicates used for this particular group. Likewise, the efficiency in the transfection of the cells used for the immunofluorescence experiments might also be affected. Another limitation to be considered is the use of solely one technique for the assessment of ROS generation and oxidative stress. Future studies should corroborate these findings using additional approaches. Despite using two independent assays to detect cellular senescence, SA‐β‐gal results should be interpreted with caution, as increased intensity of the staining in some cells did not always correlate with an extension of the cytoplasm, which is a well‐known characteristic of senescent cells. Therefore, SA‐β‐gal staining and higher levels of tumor suppressor proteins may not always indicate a permanent cell cycle arrest, but probably a stress‐induced transient cell cycle arrest that might trigger alternative processes such as apoptosis or autophagy. This assumption fits well with our data, where miR‐24 overexpression and Prdx6 downregulation lead to cell death and increased senescence. Alternatively, the cells overexpressing miR‐24 or cells with downregulated levels of Prdx6 may enter irreversible senescence and the final post‐senescent stage of cell death (Gamez et al., 2019). Cell senescence and cell death share common factors and both have been shown to be interdependent in certain scenarios (Gamez et al., 2019). Our data indicate that overexpression of miR‐24 and downregulation of Prdx6 are associated with changes in mitochondrial morphology, increase in mitochondrial ROS generation, and increased levels of phospho‐H2Ax (Figures 3,6). It has been shown that following DNA damage, cells undergo a temporary cell cycle arrest in an attempt to repair their DNA; if the DNA damage is unresolved, cells can undergo apoptosis or become senescent; in case of increased damage in senescent cells, senescent cells may undergo cell death (Gamez et al., 2019).

Noteworthy, myogenic progenitors isolated from mouse were from males, whereas human samples were retrieved from female donors. Several studies have shown biological differences between rodent males and females in the development of sarcopenia and efficiency in muscle regeneration, as well as in the global expression of microRNAs in human skeletal muscle (Kob et al., 2015; Maher et al., 2009). It is thus important to point out that the altered expression of genes in satellite cells and muscle progenitors shown in this study might be sex‐specific in addition to species‐associated differences.

In summary, our results identify a role for miR‐24‐3p through inhibition of Prdx6 in satellite cells during aging which may play a key role in early stages of skeletal muscle regeneration after acute injury, through controlling adaptive redox and apoptotic and senescence signaling pathways. Moreover, our findings show that miR‐24 and Prdx6 regulation of myogenic progenitor phenotype is more pronounced in cells from old mice, likely due to miR‐24 regulation of additional to Prdx6 target genes, such as p21. This mechanism may not be as strongly conserved in mice as in humans, as the effects of miR‐24‐regulated mitochondrial ROS, myoblast viability, differentiation and senescence were more pronounced in myoblasts from adult humans. This is not surprising, as miR‐24 binding site in Prdx6 resides at the 3’UTR of the human Prdx6 transcript, whereas in mice, this site has a weaker interaction at the 5’UTR of the Prdx6 transcript. The role of miR‐24 in the regulation of muscle regeneration requires further in vivo studies given the subtle differences in the phenotype induced by miR‐24 on myogenic progenitors from adult and old mice, as these could be further exacerbated through changes in the satellite cell niche during aging.

We propose that changes in miR‐24 and Prdx6 levels in satellite cells during aging represent an adaptive response to aging aimed at improving cellular viability and myogenic potential and decrease of cellular senescence through regulating mitochondrial ROS generation and potentially associated with it DNA damage.

## 4 EXPERIMENTAL PROCEDURES

### 4.1 Reagents

All reagents are listed in supplementary tables.

### 4.2 Mouse samples

All experiments described herein received the ethical approval from The University of Liverpool Animal Welfare and Ethical Review Body (AWERB) and were performed in accordance with UK Home Office guidelines under the UK Animals (Scientific Procedures) Act 1986. All mice were male wild‐type C57Bl/6 from Charles River (Margaret), maintained under SPF conditions, and fed *ad libitum* and maintained under barrier on a 12‐hour light/dark cycle. For muscle regeneration, tibialis anterior muscle was injured by intramuscular injection of barium chloride (1.2% in saline). Tissue was collected 1, 7, 14, or 21 days after injury. Muscle was snap‐frozen in liquid nitrogen and stored at −80°C. Muscle progenitor cells and satellite cells were directly isolated from fresh lower limbs muscles (extensor digitorum longus, tibialis anterior, gastrocnemius, quadriceps, and soleus). For each experiment, *n* = 3–7 independent replicates per group were used. Young: 6–12 weeks old; adult: 6–8 months old; old: 20–24 months old. For miR‐24 and Prdx6 expression in FACS‐sorted satellite cells: adult: 1–8 months old; old: 20–24 months old.

### 4.3 WGA staining

Wheat germ agglutinin (WGA) staining was performed on snap‐frozen cryosection samples of gastrocnemius muscles. Sections were briefly fixed for 10 min on ice‐cold methanol, washed twice with PBS 0.04% tween‐20 (PBST), and incubated for 10 min with 1:1000 WGA conjugated to Fluorescein (VectorLab, FL‐1021) in PBS. Sections were rinsed with PBS three times and were next incubated with DAPI (1:10,000, Sigma, D9542). Next, sections were mounted in Fluoromount‐G (SouthernBiotech, 0100–10) and visualized.

### 4.4 Human samples

All experiments described herein involving human samples were performed according to good practice guidance and in accordance with The University of Liverpool, University Hospital Aintree Hospital and South West Wales Research Ethics Committee (Approval No: 13/WA/0374). The University of Liverpool acted as the ethics sponsor for this study. All the donors had given informed consent for enrollment in this study. Muscle biopsies were obtained from foot surgeries (extensor digitorum brevis, tibialis anterior, or abductor hallucis muscles) of female patients treated for Hallux Valgus, with an average age of 33 ± 6.78 years old and a body mass index (BMI) <25. For each experiment, and due to limitations in sample availability, both human primary myogenic progenitors isolated from female donors (*n* = 2–5 per experiment) and commercialized human primary skeletal muscle progenitors (ThermoFisher Scientific, *n* = 1–2 per experiment) were used. For all the experiments *n* = 3–7 independent replicates per group, unless stated otherwise.

### 4.5 Satellite cell isolation

Satellite cells were isolated using FACS as previously described (Soriano‐Arroquia et al., 2016; Yi & Rossi, 2011). Briefly, skeletal muscle was isolated from the hind limbs of C57Bl/6 wild‐type male mice and enzymatically digested with 1.5 U ml^‐1^ collagenase D, 2.4 U ml^‐1^ dispase II, and 2.5 mM CaCl_2_. Cells were then dissolved in sterile FACS buffer (2% horse serum in DPBS), filtered through a 40‐µm cell strainer, and stained with conjugated antibodies in the dark for 30 min on ice. Doublets were discriminated, and hematopoietic and endothelial cells (PE‐CD31^+^/CD45^+^) were excluded from the sorting gates. Satellite cell population was isolated as BV421‐CD34^+^, Alexa647‐Alpha7Integrin^+^, FICT‐Sca1^−^, PE‐CD31^−^, PE‐CD45^−,^ and eFluor780‐Viability^−^ dye. Sorting was performed at 4°C and samples were collected in growth media (high‐glucose DMEM supplemented with 10% FBS, 1% L‐glutamine, and 1% penicillin/streptomycin). Sorted cells were immediately centrifuged and resuspended in Qiazol (Qiagen) for total RNA isolation.

### 4.6 RNA isolation

For RNA isolation, cells were collected 48 h after transfection. Total RNA from sorted cells was isolated using miRNeasy Mini Kit (Qiagen). Total RNA from primary cells was isolated using TRIzol/chloroform standard protocol. After isolation, if necessary samples were purified using ethanol and sodium acetate, RNA concentration and quality were assessed using Nanodrop 2000.

### 4.7 Real‐Time qPCR

cDNA synthesis and real‐time qPCR were performed as previously described (Soriano‐Arroquia et al., 2016). Briefly, cDNA synthesis was performed from 500 ng of RNA (for mRNA) or 100 ng of RNA (for microRNA) using SuperScript II (ThermoFisher) or miRscript RT kit II (Qiagen), respectively. SYBR Green Mastermix (Qiagen) or SsoAdvanced Universal SYBR Green Supermix (BioRad) or FastSybrGreen (ThermoFisher; 4385610) were used for real‐time quantitative PCR. Relative expression to β‐actin, 18S, S29, β‐2 microglobulin (mRNA), or Snord‐61 (microRNA) was calculated using delta C_t_ method (Soriano‐Arroquia et al., 2016).

### 4.8 Isolation of primary muscle progenitor cells from mouse and human skeletal muscles

The isolation of human and mouse primary muscle progenitor cells was performed as previously described (Soriano‐Arroquia et al., 2017). Briefly, skeletal muscle tissue was enzymatically digested with 1.5 U ml^−1^ collagenase D, 2.4 U ml^−1^ dispase II, and 2.5 mM CaCl_2_. Digested muscles were harvested on culture dishes coated with 10 µg ml^−1^ laminin and cultured with F‐12 media complemented with 20% FBS, 10% horse serum, 1% L‐glutamine, 1% penicillin/streptomycin, and 2.5 ng/ml bFGF (recombinant human FGF‐basic). Human cells were grown in high‐glucose DMEM supplemented with 20% FBS, 10% horse serum, 1% L‐glutamine, and 1% penicillin/streptomycin, and mouse cells were grown in high‐glucose DMEM supplemented with 10% FBS, 1% L‐glutamine, and 1% penicillin/streptomycin. For differentiation, both human and mouse primary muscle progenitor cells were cultured in high‐glucose DMEM supplemented with 2% horse serum, 1% L‐glutamine, and 1% penicillin/streptomycin.

### 4.9 Transfections and immunostaining

All cells in main figures were isolated from 6‐month (adult) or 24‐month (old)‐old mice. Cells used in experiments presented in supplementary data were isolated from mice aged 1–8 months (young and adult) or 20–24 months (old). Transfections of primary cells were performed as previously described (Soriano‐Arroquia et al., 2017). Briefly, primary cells were transfected with 100 nM of miR‐24‐3p mimic, 100 nM of miR‐24‐3p inhibitor, 100 nM of scrambled control, or 100 nM of siRNA against Prdx6 using Lipofectamine 2000 transfection reagent (ThermoFisher). Cells were used at P4‐P7 (or P7‐P10 in preliminary data). Cells were plated at either 80% confluency (differentiation, qPCR, Western blotting) or 50% confluency (viability, proliferation, senescence, MitoTracker, and MitoSox staining). Culture media were changed to differentiation media (high‐glucose DMEM complemented with 2% HS, 1% P/S, 1% α‐glutamine) 6 h after transfection. No media were changed until collection or staining of the cells. Control cells were transfected with scrambled control. Immunostaining was performed 48 h (Ki67 staining), 4 days (viability assay), 7 days (SA‐β‐galactosidase staining), and 7–10 days (MF 20 staining) after transfection. RNA and protein were isolated 48 h after transfection. Staining for MF 20, SA‐β‐galactosidase, MF 20, Ki67, and viability assay were performed as previously published methods (Soriano‐Arroquia et al., 2017). Fluorescent SA‐β‐galactosidase, MitoTracker Red, MitoSox, and DNA damages were performed using Cell Event Cell senescence kit, MitoSox Red, MitoTracker Red CM‐H2Xros, and HCS DNA damage kits (ThermoFisher) according to manufacturer's protocols.

For Western blotting, cells were lysed in RIPA buffer and protein concentrations were calculated using Bradford reagent with BSA as standards. For immunoblotting 20 µg (mouse) or 15 µg (human) of protein was loaded on a 10%–14% polyacrylamide gels. Following gel electrophoresis, samples were transferred onto nitrocellulose membrane and total protein was stained using Ponceau S. Following washing of the membrane with TBS‐T, membranes were blocked for 1 h at room temperature using either 5% BSA or milk, and membranes were washed 3 × 10 min in TBS‐T and incubated overnight at 4°C with primary antibodies (see supplementary tables). Membranes were washed and incubated with secondary antibodies goat antirabbit and goat antimouse (Li‐Cor Biosciences), and images were obtained using Odyssey Fc imaging system (Li‐Cor). Quantification of blots and normalization was performed using Image Studio Lite (Li‐Cor).

### 4.10 miR: target binding reporter assay

5′UTR of Prdx6‐202 transcript regions with either the wild‐type or mutated miR‐24‐3p target sites was synthesized using GeneArt service (Thermo Scientific). The wild‐type or mutated sequences were subcloned into a GFP TOPO vector (Thermo Scientific). C2C12 myoblasts were cultured in 96‐well plates and transfected using Lipofectamine 2000™ (Thermo Scientific) with either 200 ng of the wild‐type or mutant sensor and with either 100 nM of the miR scrambled control or 100nM miR‐24 mimic. Each experiment was carried out using at least two independent plasmid preparations in triplicate. GFP fluorescence was measured 48 h following transfections using FLUOstar Optima microplate reader (BMG Labtech).

### 4.11 Image analysis

Cells were semi‐automatically quantified using Fiji and ImageJ (Schindelin et al., 2012) followed by manual correction. At least 3–6 random images from different fields of view per biological sample at 10× magnification (100× total magnification) were captured. The only exception of this rule was for human β‐galactosidase analysis, in which a complete tiled field of view image was analyzed per biological sample. For myogenic differentiation analyses, fusion index is shown as the percentage of nuclei contained within myotubes to the total number of nuclei in each field of view. For the quantification of senescent cells, cells were counted manually (only cells showing intense blue staining were classified as senescent) or β‐galactosidase activity values (BGAVs) were calculated as previously described by Shlush et al., 2011. (Shlush et al., 2011). Cells with a BGAV ≥15 were considered as highly senescent (SA‐βgal^high^, characterized by an intense blue staining); cells with a BGAV between 5 and 14 both inclusive were considered as low senescent (SA‐βgal^low^, characterized by an light blue staining); and cells with a BGAV <5 were considered as non‐senescent (SA‐βgal^non^, no blue staining). All the immunostaining quantifications were manually curated. Images were captured using Nikon Eclipse Ti‐E inverted confocal microscope (supplementary data) and Carl Zeiss Axiovert 200 inverted microscope (for SA‐βgal staining) or EVOS M5000 and EVOS M7000 (ThermoFisher, main figures).

### 4.12 Statistical analysis

Details of the statistical analyses used per experiment are described in the corresponding figure legend. *T* test or Mann–Whitney (qPCR data which are not normally distributed) test was performed for the analysis of statistical differences between two groups as stated. One‐way or two‐way ANOVA followed by Tukey's multiple comparison test or Kruskal–Wallis followed by Dunn's multiple comparison test with 95% confidence Interval was performed to compare more than two groups as indicated where data were not normally distributed. *p*‐value < 0.05 was considered statistically significant. All analysis was performed on raw (not normalized) data. For the transfection experiments, individual values representing the same independent biological replicate have been matched with a dotted line. Statistical analysis was performed using GraphPad Prism version 8.4.2/9.0.0 for Windows (GraphPad Software, www.graphpad.com).

### 4.13 Gene ontology

A list of human and mouse miR‐24 predicted targets was obtained from TargetScanHuman 6.2 (Agarwal et al., 2015; Grimson et al., 2007; Lewis et al., 2005). Human and mouse miR‐24:targets network interaction and GO analyses were performed using Cytoscape v.3.8.0 (Shannon et al., 2003) and ClueGO v.2.5.6 plugin for Cytoscape (Bindea et al., 2009), respectively. Details of the statistics used for ClueGO are specified in the corresponding figure legend: generally enrichment/depletion (two‐sided hypergeometric test); minimum *p*‐value cutoff =0.01; correction method =Bonferroni step down; min GO level =5; max GO level =8; Kappa score threshold =0.4–0.55.

## CONFLICT OF INTERESTS

The authors declare no conflict of interests associated with this manuscript.

## AUTHOR CONTRIBUTIONS

AS, JG, QX, KW, BMcD performed the experiments; AS, JG, DB, QX, BMcD, and KW performed data analyses; all authors contributed to experimental design, statistical analyses, and manuscript preparation.

## Supporting information

Figures S1‐S6

Supplementary Material

## Data Availability

Source data for microscopy images and FACS data have been deposited into Mendeley dataset: https://doi.org/10.17632/g7593chtxy.1.
