# Supplementary figures and images for: miR‐24 and its target gene Prdx6 regulate viability and senescence of myogenic progenitors during aging
Source: Aging Cell. 2021 Sep 24;20(10):e13475. doi: 10.1111/acel.13475 (PMC8520721; doi:10.1111/acel.13475)

**Fig. S1**

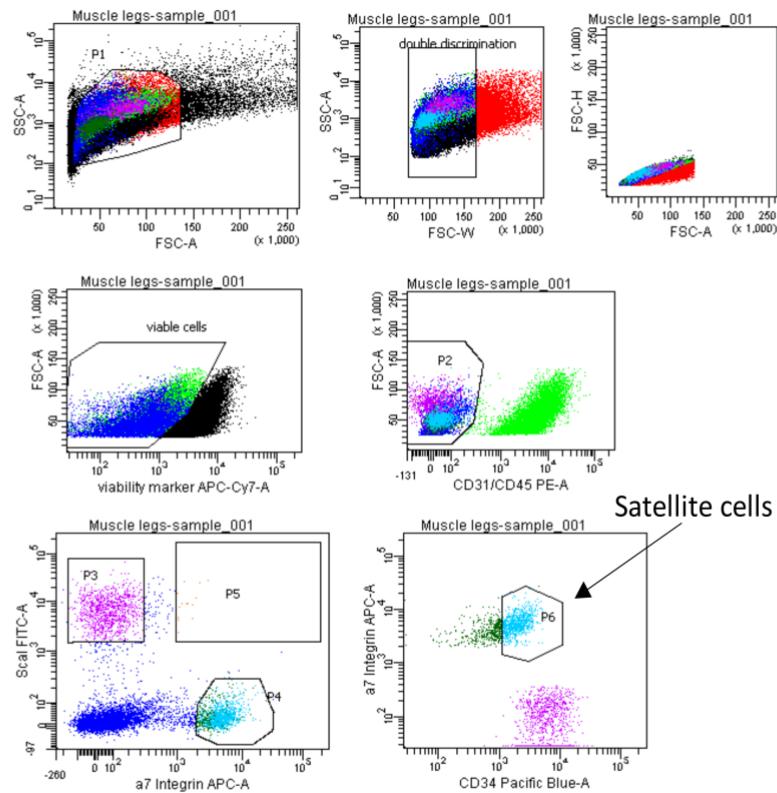

**Fig. S2**

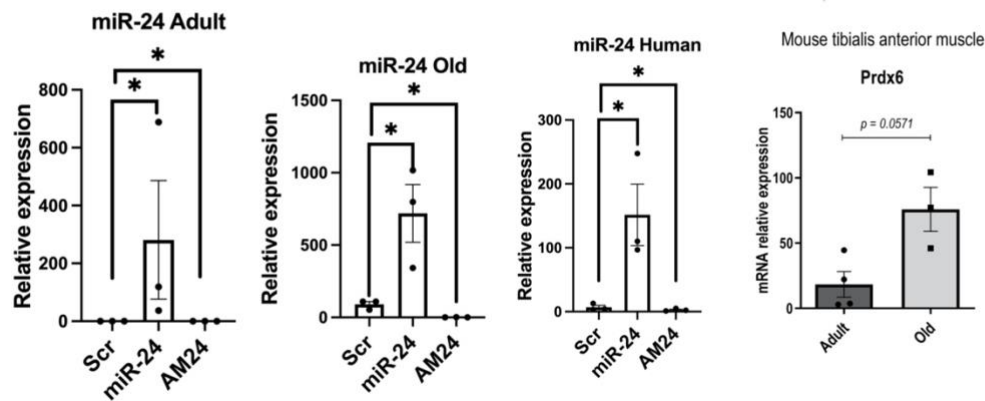

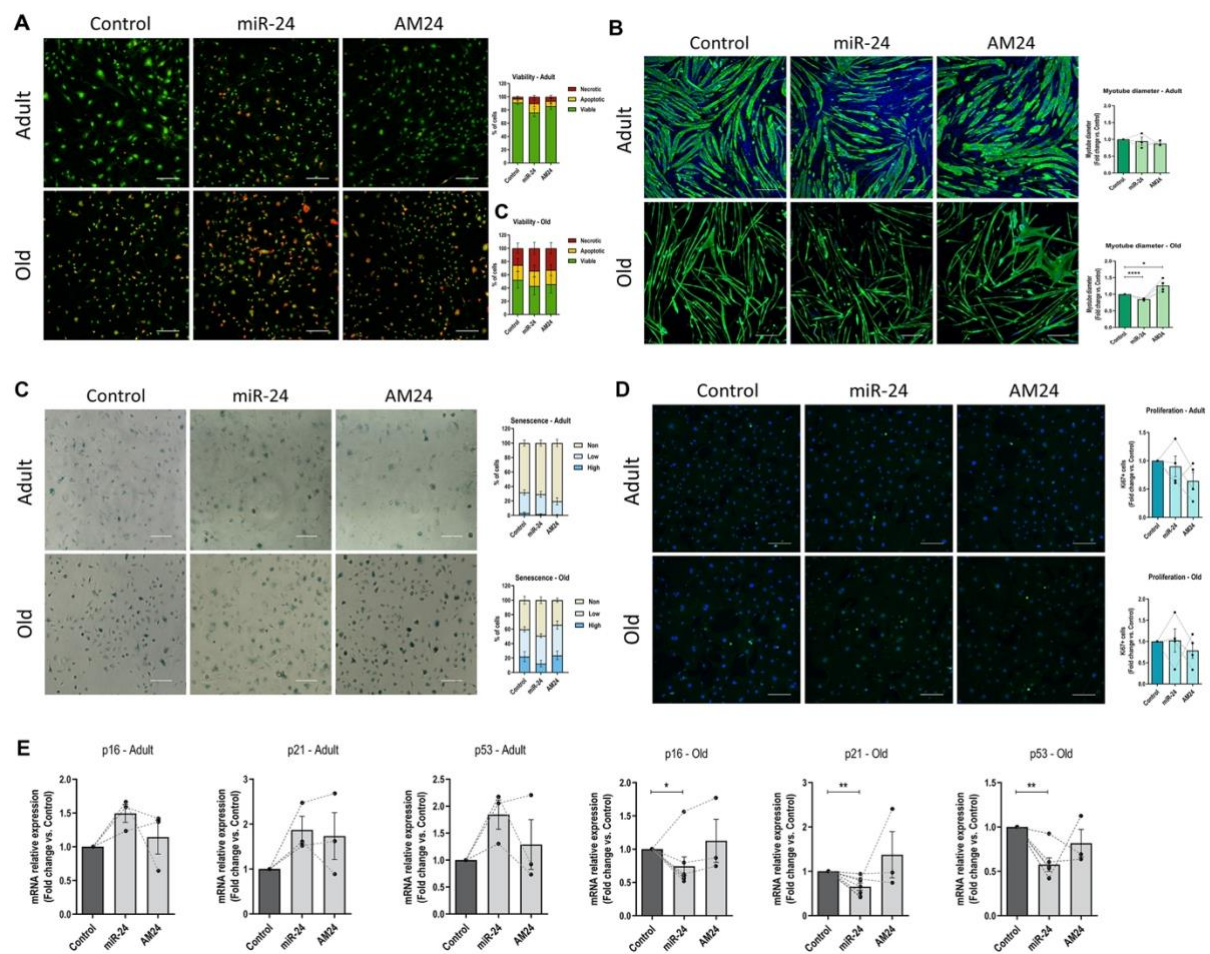

Figure S3.

# Human primary myoblasts

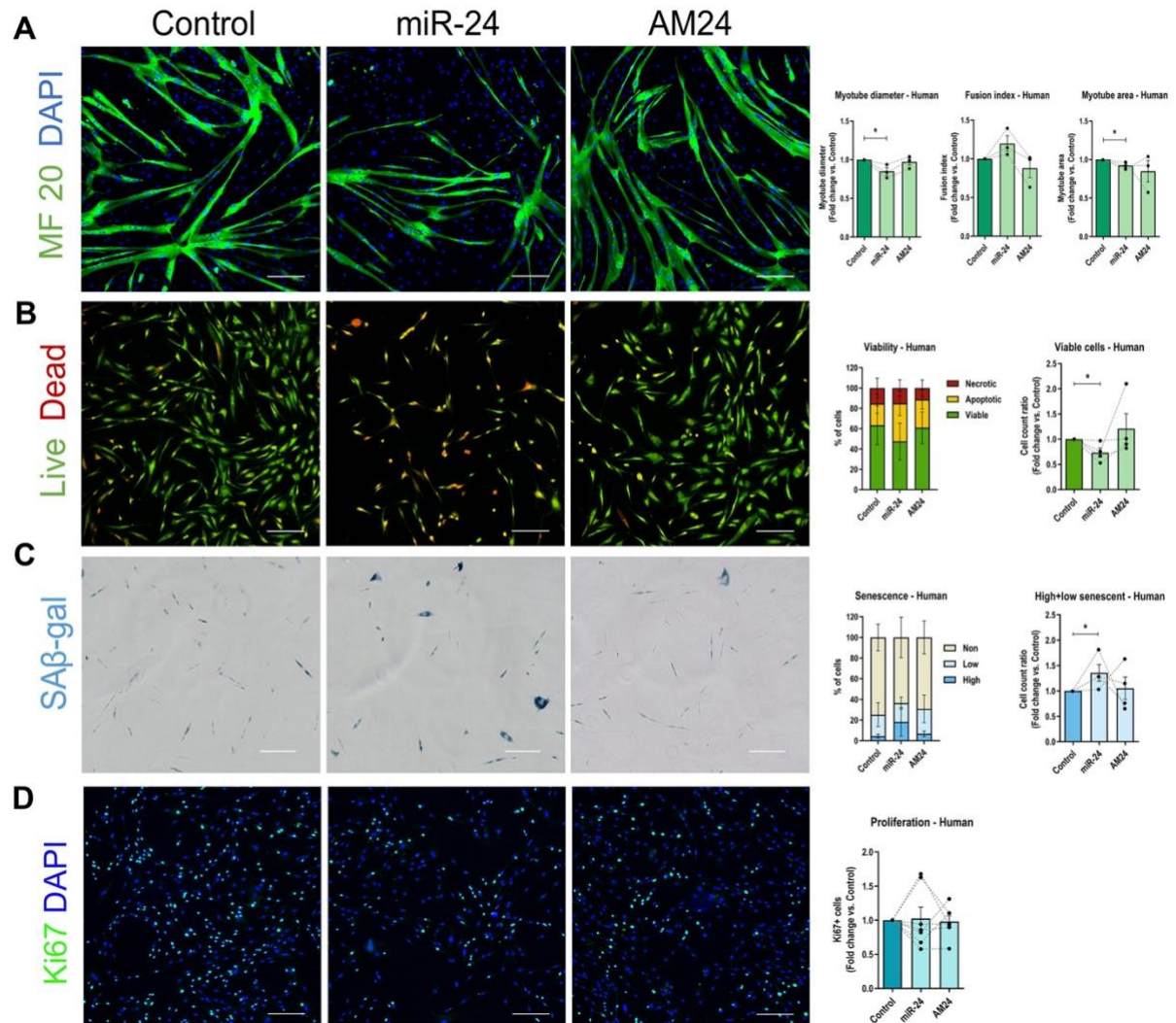

Figure S4.

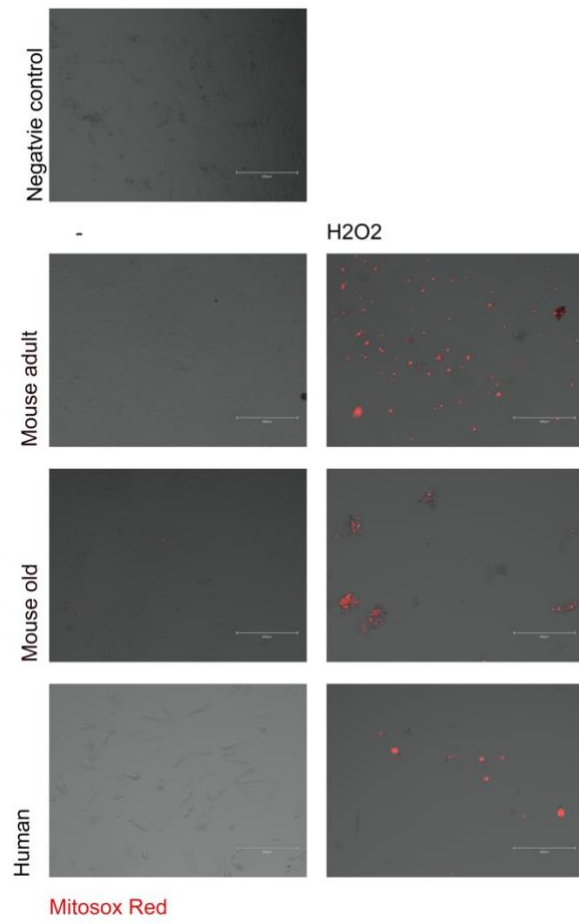

Figure S5.

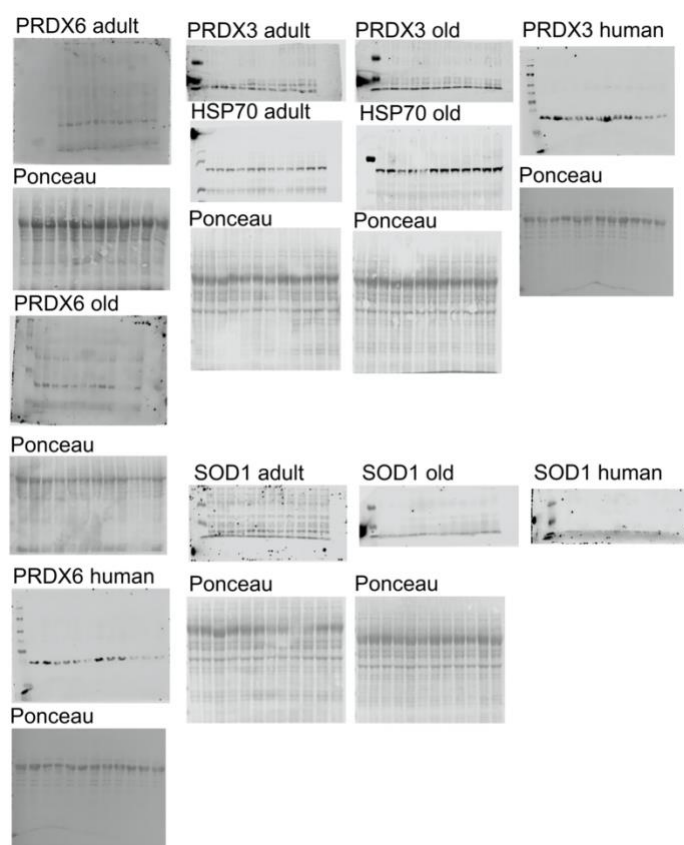

Figure S6.

Supplement: Supplementary file 1 — Figures S1‐S6 [file ACEL-20-e13475-s001.pdf]
