# Supplementary material for: miR‐24 and its target gene Prdx6 regulate viability and senescence of myogenic progenitors during aging
Source: Aging Cell. 2021 Sep 24;20(10):e13475. doi: 10.1111/acel.13475 (PMC8520721; doi:10.1111/acel.13475)
Supplement: Supplementary file 2 — Supplementary Material [file ACEL-20-e13475-s002.docx]

**Supplementary figure legends:**

**Figure S1.** Illustration showing the isolation of quiescent satellite cells by FACS (fluorescence activated cell sorting). The satellite cells population was positive for CD34, highly-positive for alpha7-integrin, and negative for Sca1, CD31 and CD45.

**Figure S2. (A)** qPCR showing the expression of miR-24 after microRNA mimic or antagomiR (AM24) transfection in primary myogenic progenitors isolated from adult and old mice. Expression relative to Snord61 is shown (n = 3-7, adult: 6-8 months old; old: 20-24 months old). F-test compared to control. p-value < 0.05 was considered as statistically significant (^#^ p < .05; ^# #^ p < .01; ^# # #^ p < .001; ^# # # #^ p < .0001). Error bars show S.E.M. **(B)** qPCR showing Prdx6 expression in mouse tibialis anterior muscle during aging. Expression relative to ß-2 microglobulin is shown (n = 3-4, adult: 6-8 months old; old: 20-24 months old). Mann-Whitney test (* < .05; **p < .01; ***p < .001). Error bars show S.E.M.

**Figure S3.** miR-24 regulates function of myogenic progenitors during aging. (A) Viability assay shows viable (green), apoptotic (yellow) and necrotic (red) cells. miR-24 overexpression resulted in decreased viable cells from both adult and old mice. miR-24 inhibition also affected viability of cells isolated from old mice (n = 6-7, two-tailed unpaired Student’s t-test compared to control). (B) MF 20 (anti-myosin heavy chain; green) and DAPI (blue) immunostaining were performed for myogenic differentiation and nuclei identification, respectively. miR-24 overexpression resulted in decreased myotube diameter whereas miR-24 inhibition resulted in bigger myotubes in myogenic progenitors from old mice compared to control (n = 3-4, two-tailed unpaired Student’s t-test compared to control). (C) SA-βgal staining performed on replicatively senescent cells from adult and old mice was performed for the assessment of senescent cells (blue). Scale bars: 200µm. Changes in miR-24 levels affected the proportion of senescent cells, (n = 4-6, two-tailed unpaired Student’s t-test compared to control). (D) Ki67 staining of primary myogenic progenitors demonstrates no difference in cell proliferation following miR-24 overexpression or inhibition. Scale bars: 200µm. n = 4-7; two-tailed unpaired Student t-Test. (E) The expression of senescence-associated genes was affected following miR-24 overexpression in myogenic progenitors from old mice. (n = 3-7, Kruskal-Wallis test followed by Dunn’s multiple comparisons test with 95% Confidence Interval; expression relative to 18S is shown). All panels: Cells transfected with scrambled RNA were used as control group (Control); adult: 6-8 months old; old: 20-24 months old; p-value < 0.05 was considered as statistically significant (*p < .05; **p < .01; ***p < .001). Error bars show S.E.M.

**Figure S4.** miR-24 regulates differentiation, viability and senescence of human primary myogenic progenitors. Human primary myogenic progenitor cells isolated from adults were transfected with miR-24 or AM24. Cells transfected with the empty vector or scrambled control were used as control (Control). **(A)** MF 20 (anti-myosin heavy chain; green) and DAPI (blue) immunostaining were performed for myogenic differentiation and nuclei identification, respectively. **(B)** Viability assay was performed with ethidium bromide and acridine orange for the assessment of viable (green), apoptotic (yellow) and necrotic (red) cells. **(C)** SA-βgal staining was performed for the assessment of senescent cells (blue). Scale bars: 200µm. **(D)** Ki67 staining indicates no effects of miR-24 on human myoblast proliferation. miR-24 overexpression resulted in thinner myotubes, less viability and increased number of senescent cells (n = 3-4, two-tailed unpaired Student’s t-test compared to Control group). p-value < 0.05 was considered as statistically significant (*p < .05; **p < .01; ***p < .001). Error bars show S.E.M.

**Figure S5.** Negative control for MitoSox Red staining and Mitosox Red staining in cells not treated or treated with H2O2 to test the working concentration od MitoSox Red.

**Figure S6.** Original western blot images.

**Supplementary table 1:** Age and gender of the patients donating a muscle biopsy for the isolation of human primary myogenic progenitor cells.

| **Donor ID** | **Age (years)** | **Gender** | **BMI** |
| --- | --- | --- | --- |
| Donor 1 | 32 | Female | <25 |
| Donor 2 | 34 | Female | <25 |
| Donor 3 | 32 | Female | <25 |
| Donor 4 | 31 | Female | <25 |
| Donor 5 | 22 | Female | <25 |
| Donor 6 | 35 | Female | <25 |

**Supplementary table 2:** Antibodies used for the isolation of satellite cells by FACS.

| **FACS Conjugated antibody** | **Company** | **Catalogue number** | **Dilution** | **FACS Aria filter** |
| --- | --- | --- | --- | --- |
| Anti-CD31-PE: PE Rat Anti-Mouse CD31. Clone: MEC 13.3. Isotype: Rat IgG2a, κ. 0.2 mg/ml | BD Biosciences PharmingenTM | 561073 | 1:1333 | 575/26 (PE) |
| Anti-CD-45/PE: PE Rat Anti-Mouse CD45. Clone: 30-F11. Isotype: Rat IgG2b, κ. 0.2 mg/ml | (BD Biosciences PharmingenTM | 553081 | 1:1333 | 575/26 (PE) |
| Anti-Sca1/FICT: FICT Rat Anti-mouse Ly-6A/E. Clone: E13-161.7. Isotype: Rat IgG2a, κ. 0.5 mg/ml | BD Biosciences PharmingenTM | 553335 | 1:1333 | 530/30 (FITC) |
| Anti-Alpha 7 Integrin 647. Clone: R2F2. Isotype: Rat IgG2b. 1.0 mg/ml | AbLab | N/A | 1:2000 | 660/20 (APC) |
| BV421 Rat Anti-Mouse CD34 Clone RAM34 (RUO). 0.2 mg/ml | BD Biosciences PharmingenTM | 562608 | 1:1000 | 450/40 (Pacific Blue) |
| Fixable Viability Dye eFluor 780 (label dead cells) | Affimetrix eBiosciences | 65-0865-14 | 1:4000 | 780/60 (APC-Cy7) |

**Supplementary table 3:** List of primers, oligos and antibodies used for the study.

| **Gene** | **Company** | **Organism** | **Sequence (5’-3’)/ Cat. Number** |
| --- | --- | --- | --- |
| Hs_SNORD61_11 miScript Primer Assay | Qiagen | Human, mouse | MS00033705 |
| Hs_miR-24_1 miScript Primer Assay | Qiagen | Human, mouse | MS00006552. Targets mature miR: UGGCUCAGUUCAGCAGGAACAG |
| Beta-actin Forward | Sigma-Aldrich | Mouse | GATCAAGATCATTGCTCCTCCTG |
| Beta-actin Reverse | Sigma-Aldrich | Mouse | AGGGTGTAAAACGCAGCTCA |
| 18S rRNA Forward | Sigma-Aldrich | Mouse | CGGCTACCACATCCAAGGAAGG |
| 18S rRNA Reverse | Sigma-Aldrich | Mouse | CCCGCTCCCAAGATCCAACTAC |
| Beta-2 microglobulin Forward | Sigma-Aldrich | Mouse | GGAGAATGGGAAGCCGAACA |
| Beta-2 microglobulin Reverse | Sigma-Aldrich | Mouse | TCTCGATCCCAGTAGACGGT |
| p16 Forward | Sigma-Aldrich | Mouse | TGGTCACTGTGAGGATTCAGC |
| p16 Reverse | Sigma-Aldrich | Mouse | GTTGCCCATCATCATCACCTGG |
| p21 Forward | Sigma-Aldrich | Mouse | ATCCAGACATTCAGAGCCACAG |
| p21 Reverse | Sigma-Aldrich | Mouse | TCGGACATCACCAGGATTGG |
| Prdx6 Forward | Sigma-Aldrich | Mouse/  Human | TTGATGATAAGGGCAGGGAC |
| Prdx6 Reverse | Sigma-Aldrich | Mouse/ Human | CTACCATCACGCTCTCTCCC |
| Tumor protein p53 Forward | Sigma-Aldrich | Mouse | CACGTACTCTCCTCCCCTCAAT |
| Tumor protein p53 Reverse | Sigma-Aldrich | Mouse | AACTGCACAGGGCACGTCTT |
| mPRDX6-202 5’UTR Forward | Sigma-Aldrich | Mouse | GCCCCGCCCACTCGGCCAGC |
| mPrdx6-202 5’UTR Reverse | Sigma-Aldrich | Mouse | AGCAACCCTCCGGGCATGGC |
| mPrdx6-202 5’UTR WT | Sigma-Aldrich | Mouse | GCCCCGCCCACTCGGCCAGCACTGATCTAGGTCTCCGCAG**GAGCC**CGC  CCGCTGCTCACTGCTGCGGCTGCGCCTCCTTGTTCTCAGCGTCACCAC  TGCCGCCATGCCCGGAGGGTTGCT |
| mPrdx6-202 5’UTR 24 MUT | Sigma-Aldrich | Mouse | GCCCCGCCCACTCGGCCAGCACTGATCTAGGTCTCCGCAG**GATCC**CGC  CCGCTGCTCACTGCTGCGGCTGCGCCTCCTTGTTCTCAGCGTCACCAC  TGCCGCCATGCCCGGAGGGTTGCT |
| Bcl-2 Forward | Sigma-Aldrich | Mouse | CTGCAAATGCTGGACTGAAA |
| Bcl-2 Reverse | Sigma-Aldrich | Mouse | TCAGGAGGGTTTCCAGATTG |
| H2ax Forward | Sigma-Aldrich | Mouse | GGCCTGTGGACAAGAGTTCTAT |
| H2ax Reverse | Sigma-Aldrich | Mouse | GCCCATTAAATCTCCCCACT |
| P53 Forward | Sigma-Aldrich | Mouse | CACGTACTCTCCTCCCCTCAAT |
| P53 Reverse | Sigma-Aldrich | Mouse | AACTGCACAGGGCACGTCTT |
| Bcl-2 Forward | Sigma-Aldrich | Human | TCGCCCTGTGGATGACTGA |
| Bcl-2 Reverse | Sigma-Aldrich | Human | CAGAGACAGCCAGGAGAAATCA |
| H2ax Forward | Sigma-Aldrich | Human | CATGTCGGGCCGCGGCAA |
| H2ax Reverse | Sigma-Aldrich | Human | GTGGCGCTGGTCTTCTTG |
| P16 Forward | Sigma-Aldrich | Human | GAAGGTCCCTCAGACATCCCC |
| P16 Reverse | Sigma-Aldrich | Human | CCCTGTAGGACCTTCGGTGAC |
| P21 Forward | Sigma-Aldrich | Human | GGCAGACCAGCATGACAGATTTC |
| P21 Reverse | Sigma-Aldrich | Human | CGGATTAGGGCTTCCTCTTGG |
| S29 Forward | Sigma-Aldrich | Mouse/ Human | ATGGTCACCAGCAGCTCTA |
| S29 Reverse | Sigma-Aldrich | Mouse/ Human | GTATTTGCGGATCAGACCGCT |

**Supplementary table 4:** Table of reagents used for the experiments.

| **Product** | **Company** | **Catalogue number** |
| --- | --- | --- |
| Barium chloride | Sigma-Aldrich | 202738 |
| AllStars Negative Control siRNA Print  miRIDIAN Scr control | Qiagen  Dharmacon | 1027280 (discontinued)  IN-122262-00-70 |
| Syn-mmu-miR-24-3p | Qiagen | MSY0000219 |
| Anti-mmu-miR-24-3p  Anti-miR-24-3p | Qiagen  Dharmacon | MIN0000219 (discontinued)  IH-122261-00-70 |
| Mouse Prdx6 siRNA | ThermoFisher Scientific | s62375 (discontinued),  s62376 |
| Human Prdx6 siRNA | ThermoFisher Scientific | s18428  s18429 |
| Lipofectamine 2000 | ThermoFisher Scientific | 11668019 |
| MF20 primary antibody. Antigen: myosin, sarcomere (MHC). 211 ug/ml | Developmental Studies Hybridoma Bank | MF20-c 2ea |
| Rabbit mAb to Ki67 [SP6]. | Abcam | ab16667 |
| Goat anti-Mouse IgG (H+L) Secondary Antibody, Alexa Fluor 488 conjugate. | Invitrogen | A-11029 |
| Goat anti-Rabbit IgG (H+L) Secondary Antibody, Alexa Fluor 488 conjugate. | ThermoFisher Scientific | A-11034 |
| DAPI (4',6-Diamidino-2-Phenylindole, Dihydrochloride) | Sigma-Aldrich | D9542 |
| Senescence β-Galactosidase Staining Kit  Cell Event Cell senescence kit | Cell Signaling Technology  Thermo Fisher | 9860  C10850 |
| Acridine Orange hydrochloride solution, 10 mg/mL in H_2_O | Sigma-Aldrich | A8097 |
| Ethidium bromide solution. BioReagent, for molecular biology, 10 mg/mL in H_2_O | Sigma-Aldrich | E1510 |
| Methanol | Fisher | M/4000/PC17 |
| PBS (immunostaining) | Sigma-Aldrich | P4417 |
| Tween-20 | Sigma-Aldrich | P1379 |
| Wheat Germ Agglutinin (WGA), Fluorescein | Vector Laboratories | FL-1021 |
| Fluoromount | ThermoFisher | 00-4958-02 |
| DAPI (4',6-Diamidino-2-Phenylindole, Dihydrochloride). 1mg/ml | Sigma-Aldrich | D9542 |
| miRNeasy Mini Kit | Qiagen | 217004 |
| TRIzol Reagent | Life Technologies | 15596-018 |
| Chloroform:Isoamyl alcohol 24:1 | Sigma-Aldrich | C0549 |
| Isopropanol | Sigma-Aldrich | I9516 |
| RNAse-free water | Sigma-Aldrich | 3098 |
| Sodium acetate | Sigma-Aldrich | S2889 |
| Nanodrop 2000 | ThermoFisher Scientific | N/A |
| Superscript II Reverse Transcriptase | Life Technologies | 18064 |
| Random Hexamers (50 μM) | ThermoFisher | N8080127 |
| 25X dNTP Mix (100 mM) | ThermoFisher | 4368814 |
| RiboLock RNase Inhibitor (40 U/μL) | ThermoFisher | EO0381 |
| miRScript RT II | Qiagen | 218161 |
| miRScript SybrGreen PCR Kit | Qiagen | 218073 |
| T100 Thermal Cycler | Bio-Rad | 1861096 |
| CFX Connect Real-Time PCR Detection System | Bio-Rad | 1855201 |
| RNU-6 qPCR primer | Qiagen | MS00033740 |
| Snord-61 qPCR primer | Qiagen | MS00033705 |
| miR-24_1 miScript Primer Assay | Qiagen | MS00006552 |
| Select agar | Sigma-Aldrich | A5054 |
| MyTaq Red Mix | Bioline | BIO-25043 |
| GeneJET Genomic DNA Purification Kit | Thermo Scientific | K0721 |
| One Shot TOP10 Chemically Competent E. coli | Invitrogen | C404010 |
| SYBR Safe DNA Gel Stain | Invitrogen | S33102 |
| DNA Gel Loading Dye (6X) | Thermo Scientific | R0611 |
| UltraPure Agarose | Invitrogen | 16500500 |
| GFP Tag Antibody, ABfinity Rabbit Monoclonal | ThermoFisher Scientific | G10362 |
| CM-H2DCFDA (General Oxidative Stress Indicator) | Invitrogen | C6827 |
| FLUOstar OPTIMA microplate reader | BMG Labtech | N/A |
| Hydrogen peroxide solution 30 % (w/w) in H2O, contains stabilizer | Sigma-Aldrich | H1009 |
| 1x RBC (Red Blood Cell) Lysis Buffer | eBioscience | 00-4333-57 |
| FACS Aria III Flow Cytometer | BD Biosciences | N/A |
| MitoSox Red | Thermo Fisher | M36008 |
| MitotTracker Red CM-H2Xros | Thermo Fisher | M7513 |
| HCS DNA damage kit | Thermo Fisher | H10292 |
| C1 confocal laser scanning microscope system. 10x magnification. | Nikon | N/A |
| Axiovert 200 inverted microscope. 10x magnification. | Carl Zeiss | N/A |
| EVOS M5000 | Thermo Fisher | N/A |
| EVOS M7000 | Thermo Fisher | N/A |
| SOD1 | Abcam |  |
| HSP70 | Abcam | Ab181606 |
